# Next-generation antigen-presenting cell immune therapeutics for gliomas

**DOI:** 10.1172/JCI163449

**Published:** 2023-02-01

**Authors:** Catalina Lee-Chang, Maciej S. Lesniak

**Affiliations:** 1 Department of Neurological Surgery, Feinberg School of Medicine, Northwestern University, Chicago, Illinois, USA.; 2 Malnati Brain Tumor Institute, Chicago, Illinois, USA.

## Abstract

Antigen presentation machinery and professional antigen-presenting cells (APCs) are fundamental for an efficacious immune response against cancers, especially in the context of T cell–centric immunotherapy. Dendritic cells (DCs), the gold standard APCs, play a crucial role in initiating and maintaining a productive antigen-specific adaptive immunity. In recent decades, ex vivo–differentiated DCs from circulating CD14^+^ monocytes have become the reference for APC-based immunotherapy. DCs loaded with tumor-associated antigens, synthetic peptides, or RNA activate T cells with antitumor properties. This strategy has paved the way for the development of alternative antigen-presenting vaccination strategies, such as monocytes, B cells, and artificial APCs, that have shown effective therapeutic outcomes in preclinical cancer models. The search for alternative APC platforms was initiated by the overall limited clinical impact of DC vaccines, especially in indications such as gliomas, a primary brain tumor known for resistance to any immune intervention. In this Review, we navigate the APC immune therapeutics’ past, present, and future in the context of primary brain tumors.

Presentation of tumor-associated antigens (TAAs) via the major histocompatibility complex (MHC) class I and II (human leukocyte antigen [HLA] in humans) is fundamental for building a robust immune response and assuring the success of immunotherapies, including immune checkpoint blockade and immune cell–based immunotherapies (1, 2). MHC class I–mediated presentation of antigens (Ags) is fundamental to activation of granzyme- and perforin-producing cytotoxic CD8^+^ T cells. This process is vital for cytotoxic CD8^+^ T cells to target and kill undesirable cells such as virus-infected or cancerous cells. To promote an effective antitumor response, TAAs should be taken up and cross-presented by professional antigen-presenting cells (APCs), primarily dendritic cells (DCs), for the priming of naive CD8^+^ T cells (3). Subsequently, the TAA must be directly presented by tumor cells for recognition and killing by primed CD8^+^ T cells. Tumors develop multiple immune evasion mechanisms and reduce Ag presentation, including suppression of DC function and downregulation of HLA-I expression by tumor cells (4). Activation of CD4^+^ T cells by MHC class II (5), expressed preferentially by professional APCs such as DCs, macrophages, or B cells, also plays a fundamental role in mounting a therapeutic antitumor immune response. CD4^+^ T cells in brain tumors are best known for their protumoral effect driven by regulatory Foxp3^+^ T cells. However, effector CD4^+^ T helper cells can promote cytotoxic CD8^+^ T cell function via activation of DCs and regulate the myeloid compartment and tumor cells via secretion of immunomodulatory factors such as IFN-γ and TNF-α. In addition, CD4^+^ T helper cells can modulate the antitumoral humoral response by inducing plasmablast differentiation. CD4^+^ T cells are necessary to build a humoral response against tumor Ags by providing help via CD40 ligand signaling to CD40 on B cells to drive their differentiation and maturation into affinity-matured, class-switched plasma cells (6). Figure 1 summarizes MHC class I and II–mediated T cell activation and subsequent T cell subset differentiation.

## APCs in gliomas

High-grade malignant glioma and glioblastoma (GBM) are aggressive types of primary brain tumors that are almost universally fatal despite some progress in treatment and management. Most therapeutic benefit has been gained when the upfront treatment includes maximal safe resection followed by adjuvant multimodality chemotherapy (temozolomide) and radiotherapy. In clinical trials, the median progression-free survival is 5 to 7 months, and the median survival is 15 to 20 months. Better treatments and a more sustained efficacy are needed (7). These tumors are characterized by poor lymphocytic infiltration and a microenvironment preferentially populated by myeloid cells (8). Tumor-associated myeloid cells form a large and heterogeneous population of cells, including brain-resident microglia and bone marrow–derived macrophages, neutrophils, and DCs. Tumor-associated myeloid cells represent the primary APC compartment. Nonmyeloid cells such as B cells can rarely infiltrate gliomas and act as APCs. This section will briefly discuss the APC function of tumor-associated myeloid cells, DCs, and B cells and how gliomas inhibit their immune activation functions.

### Macrophages and monocytes.

Bone marrow–derived myeloid cells, including macrophages and monocytes, represent the major immune cells infiltrating gliomas (9, 10). They have a vast immune and nonimmune effector function that ranges from thrombosis, phagocytosis, and debris clearance to Ag presentation and immunosuppression (11, 12). Initiation of the processing of TAAs to subsequently present to T cells via their MHC depends on the ability of macrophages and monocytes to engulf tumor cells. The interactions between tumor cells and macrophages/monocytes that regulate this engulfment are driven by “eat me” ligands, such as calreticulin, SLAMF7, opsonizing antibodies, or phosphatidylserine, and “don’t eat me” ligands, such as CD47, PD-L1, or MHC I (13). Tumor cells use this “don’t eat me” network to prevent phagocytosis, ensure their survival, and avoid antitumor T cell response. In addition, preclinical models of GBM have shown a limited impact of Ag presentation and Ag-specific T cell expansion by macrophages and monocytes (14). In GBM, myeloid cells are best known for their immunosuppressive function driven by a multifactorial network able to shut down the antitumor adaptive immune system. This network includes immunomodulatory factors (IL-10, TGF-β, IDO-1) (15–20), metabolic remodeling of the tumor microenvironment via the arginine pathway (21), and expression of suppressive molecules such as PD-L1 (22). Seminal work led by M. Suvà and I. Tirosh (23, 24), which explored intratumoral GBM diversity, unveiled the association of tumor-infiltrating macrophages with the mesenchymal-like (MES-like) state, one of the four malignant cellular states that define GBM heterogeneity. Further analysis of this association revealed a reciprocal interaction and underlined how macrophages induce the MES-like state via the secretion of oncostatin M. This work highlights the direct gliomagenesis effect of tumor-associated macrophages, which suggests that macrophages are plastic and multifunctional, and their role in supporting tumor growth goes beyond the well-documented immunosuppression.

### Neutrophils.

Like macrophages and monocytes, neutrophils are tumor-infiltrating myeloid cells that can act as APCs upon maturation. In lung adenocarcinomas and squamous cell carcinomas, these APC-like neutrophils stimulate the proliferation of both CD4^+^ and CD8^+^ T cells in an MHC-dependent manner, and stimulate expression of the costimulatory molecules CD86, 4-1BB ligand, and OX40 ligand (25, 26). Secretion of lymphocyte-chemoattractant factors such as CXCL10, CCL2, CCL3, CXCL1, and CXCL2 further amplifies the potential of neutrophils to impact T cell immunity (27, 28). Both macrophages and neutrophils are highly susceptible to the immunosuppressive microenvironment of gliomas. For instance, gliomas recruit macrophages and monocytes (29) and rapidly convert them into glioma-supportive cells. This process involves the generation of specific metabolic niches, such as hypoxia (30, 31), or the production of metabolites with immunosuppressive capabilities, such as polyamines (21). Also, mutated IDH1 gliomas secrete immunomodulatory factors such as G-CSF that can inhibit the positive immune response of neutrophils (32). Because of the immunosuppressive and tumorigenic effect of glioma-associated macrophages and neutrophils, their inhibition or depletion are attractive therapeutic approaches (22, 32).

### Microglia.

Microglia are resident myeloid cells of the central nervous system (CNS) with a known capacity to present Ags and activate cytotoxic T cells (33, 34). However, the immunosuppressive microenvironment of gliomas downregulates MHC expression, which limits their APC ability (35–39). Glioma cells also stimulate the secretion of IL-10 and inhibit the production of TNF-α by microglia, further promoting the suppression of the immune response (40).

### DCs.

DCs are typically not found in normal brain parenchyma but are present in the choroid plexus and meninges; this is suggestive of potential migratory pathways of peripheral DCs into the CNS (41–43). During chronic inflammatory diseases, acute infections, neurodegeneration, and cancer, DCs can migrate to the brain and spinal cord through either afferent lymphatics or high endothelial venules (44). The specific role of DCs in gliomas remains to be fully elucidated. Still, current studies suggest a complex interplay between DCs, microglia and macrophages, T cells, and tumor cells in the tumor microenvironment. One suggested role for DCs in this context is in recognizing and presenting tumor Ags in the brain or the tumor-draining deep cervical lymph nodes to elicit coordinated T cell–mediated responses (44). Through signal 1 and 2 costimulatory interactions, these DCs mobilize and stimulate the development of various effector T cells associated with immune defense, such as cytotoxic T cells and CD4^+^ T helper cells (45, 46). Indeed, the immunosuppressive milieu of gliomas is harsh on DCs. Recent explorations into the role of DCs in glioma progression have focused on homeostatic regulators of DC function, including Nrf, a redox-sensitive transcription factor that is involved in counteracting the effects of reactive oxygen species. The tumor microenvironment of GBM is thought to induce overexpression of Nrf in DCs, resulting in the suppression of DC maturation and the consequent decrease in effector T cell activation. The inhibition of Nrf2 pathways rescues the maturation of CD80^+^ and CD86^+^ DCs in a glioma-conditioned medium and partially restores the secretion of bioactive cytokines such as IL-12p70 (47).

Extracranial tumor Ag presentation occurs in peripheral lymph nodes. Activated T cells have been found in the deep cervical lymph nodes of rat GBM models (48). This activation is controlled by DCs that migrate from the CNS to the lymph nodes via the lymphatics (49). Alternatively, CNS- and tumor-associated Ags can move out of the CNS through perivascular spaces and be collected by resident DCs in cervical lymph nodes (50, 51).

### B cells.

Mature B cells recognize Ags (soluble or cell-bound Ags) using their B cell receptor (BCR) and are activated to become antibody-producing cells. As part of the differentiation to plasmacytes, B cells use the MHC class II Ag presentation pathway to process BCR-bound and internalized protein Ags and present selected peptides in complex with MHC II to CD4^+^ T cells (52). Under pathological and inflammatory conditions, B cells can also cross-present exogenous Ags to CD8^+^ T cells via their MHC I (53, 54). The antitumor effect of B cells in cancers came from studies showing that their intratumoral density is associated with a good prognosis in breast cancer (55), colorectal cancer (56, 57), non–small cell lung cancer (58), head and neck cancer (59), ovarian cancer (60), biliary tract cancer (61), primary cutaneous melanoma (62), metastatic melanoma (63), and hepatocellular carcinoma (64). The analysis of 54 cohorts of 25 cancer types revealed that although the prognostic impact of tumor-infiltrating B cells was positive in 50% of the studies, it was deleterious in 9% and neutral in 41% (65). A few studies addressed the question of the role of regulatory B cells (Bregs), which have an immunosuppressive phenotype, in human cancers. The frequency of IL-10–producing Bregs correlated with shorter overall survival in bladder cancer patients (66) and in breast cancer (67), and the coexistence of Bregs with regulatory T cells correlated with shorter metastasis-free survival in breast cancer (68). These findings suggest that protumoral and antitumoral B cells might coexist. In neoadjuvant pembrolizumab (PD-1 blockade) treatment of soft-tissue sarcoma patients, the B cell signature was the best predictor of overall survival, even when combined with CD8, PD-1, or CTLA-4 signatures (69). In advanced metastatic melanoma, tertiary lymphoid structure (TLS) and B cell signatures, but not T cell signatures, predicted therapeutic responses to pembrolizumab and ipilimumab (CTLA-4 blockade). B cells in tumors of responding patients exhibited oligoclonal repertoires of the immunoglobulin (Ig) genes compared with nonresponding patients’ polyclonal B cell repertoires. Moreover, B cells and TLS densities increased during treatment only in responding patients (70). A TLS gene signature synergized with a T cell effector signature to predict responses to immune checkpoint inhibition (ICI) with PD-1 and CTLA-4 blockade (63). Antitumoral functions of B cells upon ICI therapy have been attributed to their differentiation into plasmablasts (71), the subsequent production of tumor-reactive antibodies (72–74), and T cell activation via Ag presentation (75, 76) or Ab-dependent complement activation (77). However, B cell–mediated APC immune function and T cell activation in primary brain tumors remain unclear owing to their rarity, as they represent less than 0.5% to 1% of the immune milieu (78–80). In addition to the low numbers in the tumor microenvironment, gliomas promote the conversion of B cells into immunosuppressive B cells that sustain tumorigenicity (81). More recently, work published by our groups suggested a more immunosuppressive function of glioma-infiltrating B cells, characterized by the suppression of CD8^+^ T cell activation in both GBM patients and glioma-bearing mice (79).

## APCs as cellular immunotherapy

### DC vaccines.

The primary goal of APC vaccination is to harness T cell antitumor immunity by the presentation of TAAs. DC vaccines were the first cell-based immunotherapy developed to treat cancers (82, 83). DCs in cancer immunotherapies can be explored by different approaches: (a) bulk tumor protein- and/or nucleic acid–based vaccines; (b) peptides targeting endogenous DCs; (c) ex vivo–generated DCs matured and loaded with tumor Ags; and (d) biomaterial-based platforms for the in situ recruitment and reprogramming of endogenous DCs (84, 85). Among the registered clinical trials performed with DC vaccines, the most common approach relies on the use of ex vivo–differentiated DCs from leukapheresis-isolated CD14^+^ monocytes (MoDCs) cultured in the presence of granulocyte-macrophage colony-stimulating factor (GM-CSF) and IL-4 (86). Previous studies have highlighted the effectiveness of DC vaccination for gliomas in preclinical models and early-stage clinical trials (87–89). Preclinical work in rodents has shown that DCs pulsed with tumor-derived Ags elicited strong tumor-reactive T cell immunity and could prolong glioma-bearing animal survival (87, 90). These experimental proof-of-principle approaches paved the way for the development of several autologous DC vaccines pulsed with tumor lysates as a therapeutic for treating primary brain tumors (91–94). Pioneered by Liau et al., the first DC vaccines were tested in GBM patients using acid-eluted tumor Ags (95). These initial investigations by Liau and colleagues led to a phase II clinical trial (ClinicalTrials.gov NCT00045968). This randomized trial reported that DCVax-L, in combination with standard of care, could significantly extend the survival of patients with either newly diagnosed or recurrent GBM compared with historical controls. This randomized trial reported that DCVax-L in combination with standard of care could significantly extend the survival of patients with either newly diagnosed or recurrent GBM (96). Liau’s seminal work paved the way for further development of the field of DC vaccination in GBM.

As an alternative to using whole-tumor lysates, synthetic peptides can also be used to pulse DC vaccines. ICT-107 is a patient-specific DC-based immunotherapy for newly diagnosed GBM patients. ICT-107 DCs are pulsed with six synthetic TAAs (MAGE-1, HER-2, AIM-2, TRP-2, gp100, and IL-13Rα2) instead of a bulk tumor lysate approach seen in previous studies. ICT-107 was tested in a double-blind, placebo-controlled phase II trial to evaluate its safety and efficacy when administered in conjunction with the Stupp protocol for newly diagnosed GBM (97). This trial highlighted that prolonged overall survival (OS) correlated with the expression of four ICT-107–targeted Ags. Despite this encouraging result, the OS benefit was not confirmed in the later phase II trial (98). A phase III clinical trial was scheduled but was halted before reaching its primary outcome owing to insufficient financial resources.

Mitchell, Sampson, and colleagues showed that preconditioning of the vaccination site with tetanus/diphtheria (Td) toxoid (a potent Ag recall signal) can significantly improve the lymph node homing and efficacy of tumor Ag–specific DCs (99). DCs were pulsed with cytomegalovirus phosphoprotein 65 (pp65) RNA. This study (NCT00639639) was performed on six patients with newly diagnosed GBM and showed promising results with prolonged OS in comparison with the control group that received autologous lymphocytes. A follow-up clinical trial (NCT03615404) will test this DC vaccine approach in combination with GM-CSF as an adjuvant. Table 1 lists past and current clinical trials that use DCs as therapeutics in newly diagnosed GBM.

### Monocyte vaccines.

The overall encouraging preclinical and clinical results of DC vaccination in glioma patients motivated researchers to investigate complementary ways to achieve Ag presentation and subsequent antitumor T cell function successfully. Monocyte vaccines have been efficacious in triggering antitumor CD8^+^ T cell–mediated cytotoxic responses in preclinical glioma models (100). Huang et al. showed that tumor Ag–pulsed monocytes elicited a robust immune response and outperformed bone marrow–derived DCs when administered to glioma-bearing mice (100). In this report, the authors demonstrated that Ag-loaded monocytes do not activate CD8^+^ T cells directly but rather transfer Ag to endogenous splenic CD8^+^ cDCs to cross-prime naive CD8^+^ T cells. These results are consistent with previous reports that monocyte-derived cells do not trigger CTL responses directly but rather transfer Ag to lymphoid-resident CD8^+^ cDCs in murine models of viral infection (101–103), contact sensitization (104), and Ag phagocytosis (105). The group from Duke University initiated the DEMAND study in 2022 as the translational follow-up to this research study. The trial consists of a dose escalation study of monocyte Ag carrier cells for newly diagnosed GBM patients with unmethylated *MGMT* gene promoters. This phase I clinical trial (NCT04741984) uses engineered monocytes to express cytomegalovirus protein (MT-201-BM monocyte vaccine). Tie2-expressing monocytes (TEMs) were developed following a similar approach to that used by the Duke group, but not directly linked to the APC function. In 2008, De Palma et al. (106) explored the tumor tropism of TEMs and their potential for use as a carrier of IFN-α to tumors. This approach would inhibit the angiogenesis of tumors and the activation of innate and adaptive immunity. In 2019, Genenta Science initiated a phase I/II clinical trial (NCT03866109) using a single injection of autologous TEMs engineered to produce IFN-α (Temferon) in *MGMT*-unmethylated GBM patients.

### Human umbilical vein endothelial cells.

Human umbilical vein endothelial cell (HUVEC) vaccines are less commonly investigated for the treatment of GBM. The presentation of the HUVEC Ag is believed to elicit anti-angiogenic cellular and humoral immune responses, thus inhibiting tumor growth (107–109). This is particularly important because bevacizumab has only shown limited clinical benefit in the setting of recurrent GBM (110). Thus far, clinical trials investigating HUVECs for recurrent GBM have shown them to be well tolerated and yielded encouraging early results (110, 111). Other tumor cell vaccine delivery techniques involve formalin fixation of the tumor cells before injection of the vaccine. It has been demonstrated that fixation with formalin allows for better tissue preservation, which allows for the most robust immune response against the tumor cells (112). The safety and efficacy of autologous formalin-fixed tumor vaccines (AFTVs) were tested in two clinical trials examining their use with only fractionated radiotherapy and chemoradiation in patients with newly diagnosed GBM (112, 113). Both trials demonstrated a tolerable safety profile and yielded a median OS greater than 19 months. These encouraging results prompted a prospective placebo-controlled phase IIb/III trial evaluating AFTV therapy in combination with standard chemoradiotherapy (UMIN Clinical Trials Registry UMIN10602; https://www.umin.ac.jp/ctr/). Although preliminary results confirmed the safety of AFTV therapy, this trial could not find a statistically significant difference in median progression-free survival between the two experimental arms.

### Artificial APCs.

A considerable challenge in the therapeutic vaccination of GBM with DCs or monocytes is that the immune response elicited upon treatment must overcome the extreme immunosuppressive microenvironment. Gliomas and their microenvironment are highly immunosuppressive niches that lead to successful immune evasion. DC and monocyte vaccination may require the combination of additional therapeutic strategies to overcome the adverse effects of immunosuppression and immune checkpoint regulation. To overcome this limitation of autologous APCs, investigators have developed artificial APCs (aAPCs) as an alternative for both ex vivo and in vivo induction of tumor-reactive T cell immunity (114, 115). Artificial Ag presentation is less susceptible to the immunosuppressive effect of the tumor microenvironment.

Furthermore, aAPCs are an off-the-shelf approach that overcomes the challenges of autologous cell culture strategies (114, 116). There are three subcategories of aAPCs: cellular (allogeneic and xenogeneic), acellular (liposomes, magnetic beads, polystyrene beads, and biodegradable beads), and subcellular (lipid vesicles and exosomes). Table 2 summarizes current aAPC approaches in different cancer models.

Polystyrene bead–based aAPCs coated with MHC-peptide single-chain dimers or tetramers are an approach developed to stimulate tumor-specific T cells. In the context of gliomas, this APC nanoparticle was used to expand human HA-1–specific CD8^+^ T cells and to generate IL-13Rα2–specific CD8^+^ T cells to target glioma cells (117–120). An alternative approach using a similar rationale was developed to stimulate innate mucosal-associated invariant T (MAIT) cells. The aAPC design consisted of a polystyrene bead with a 5-OP-RU–loaded MR1 tetramer complex and anti-CD28 antibody (121). Subsequently, these activated MAIT cells were efficacious at killing human glioma cell lines. A limitation of aAPCs is the lack of tissue migratory capabilities (e.g., tumor) and the inability to cross-present Ags to CD8^+^ T cells. Altogether these limitations restrict the long-term maintenance of the antitumor immune response.

## B cells, the next-generation APC immunotherapy

The B cell–based vaccine is a promising yet under-investigated approach to boosting anticancer immunity (122, 123). There are several advantages of B cells as vaccines over conventional APC vaccines, including: (a) they can act as both T cell activators and antibody producers (124); (b) mature B cells can be readily manufactured ex vivo; and (c) they have high mobility, which allows their homing to essential secondary lymphoid organs as well as tumor (125). However, not all B cells will show antitumor properties (79, 126–129), and only rare B cell subsets might be good candidates for cancer vaccines. In this section, we will navigate the possibility of using proinflammatory B cells as APC vaccines, their preclinical development, and their future translational potential.

Our research and the research of others indicate that specific B cell subpopulations hold the most potential for use in treating cancer. Specifically, studies on B cell–driven inflammation have revealed that a subset of B cells expressing the costimulatory marker 4-1BB ligand (4-1BBL, or CD137L) are especially effective in activating CD8^+^ T cell antitumor cytotoxicity. 4-1BBL is the single known ligand for 4-1BB (130), a TNF family costimulatory receptor that plays a fundamental role in activating Ag-experienced CD8^+^ T cells to establish long-term immunological memory (131, 132). Expression of 4-1BBL by B cells is achieved through B cell–mediated Ag presentation, T cell costimulation (4-1BBL and CD86), and cytokine production (TNF-α) (133, 134). This observation was further confirmed using 4-1BBL^+^ B cells from newly diagnosed glioma patients’ blood (135). It was observed that 4-1BBL^+^ B cells express proinflammatory cytokines and activation markers (TNF-α, IFN-γ, CD69, and CD86) and have a superior ability to activate autologous CD8^+^ T cells compared with 4-1BBL–negative B cells (135).

**Figure 2:**
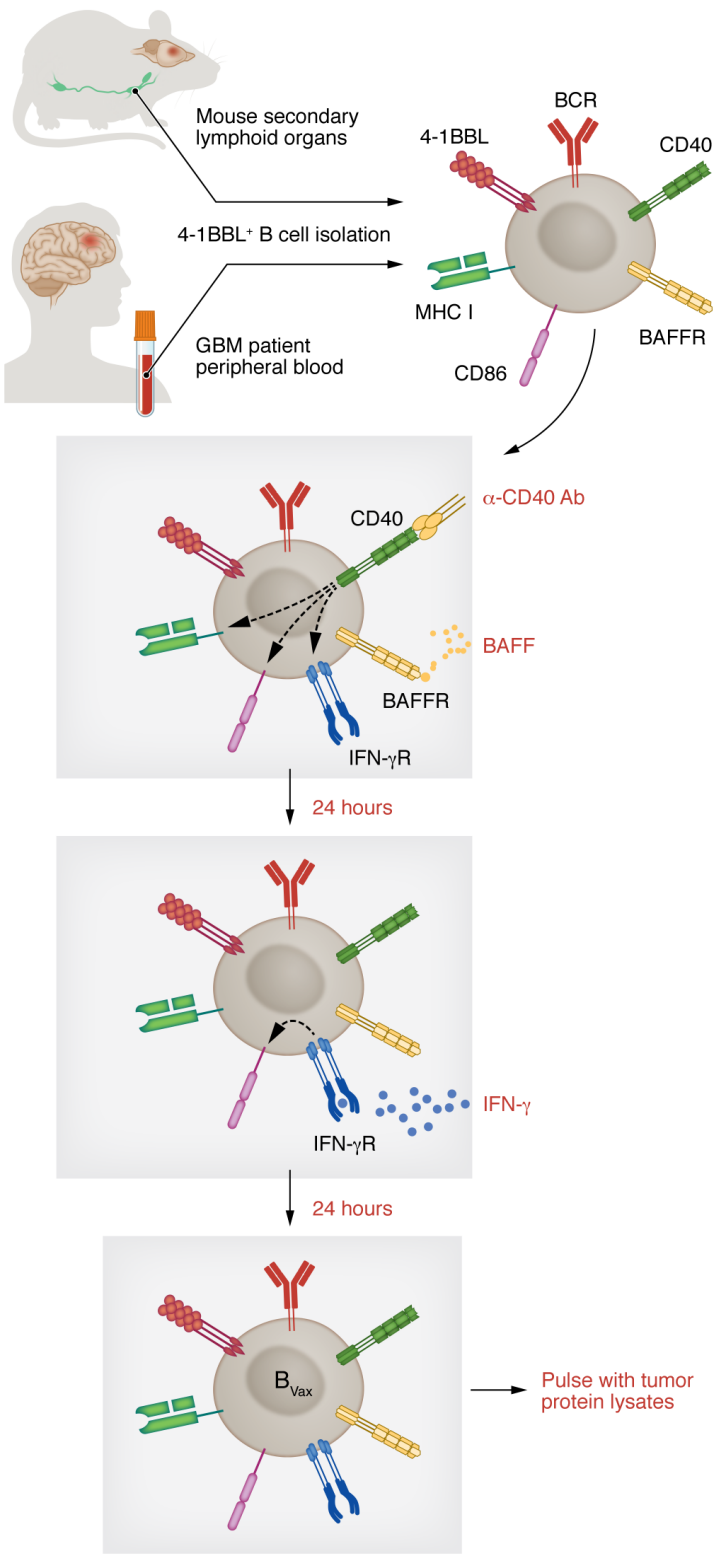
B cell vaccine generation. B cell–based vaccines (B_Vax_) are produced from 4-1BBL^+^ B cells isolated from secondary lymphoid organs of tumor-bearing mice or GBM patients’ blood. B cells are activated ex vivo using CD40 agonism, the B cell survival factor BAFF (yellow), and IFN-γ (blue). After activation, B cells are pulsed with tumor lysates.

The B cell vaccine approach used 4-1BBL^+^ activated B cells from glioma-bearing mice (secondary lymphoid organs) or GBM patient–derived PBMCs as a source of B cell–based vaccine (B_Vax_). To potentiate and stabilize the APC function, 4-1BBL^+^ B cells were further activated for a short time (48 hours) with CD40 and IFN-γ receptor (IFN-γR) activation (Figure 2) and pulsed with tumor protein lysates. CD40 ligation is a well-studied process that leads to B cell activation, proliferation, and enhancement of Ag-presenting and costimulatory functions (136), and ligand-associated activation of IFN-γR promotes the upregulation of costimulatory molecules such as CD86 in B cells (137). Unlike naive B cells, B_Vax_ could cross-present as potently as DCs in vitro. This agrees with a previous study that showed that cross-presentation by B cells activates autoimmune CD8^+^ T cells in type 1 diabetes (53). Most B cell–based vaccines use total circulating B cells (isolated using the CD20 or CD19 marker) and are activated ex vivo using CD40 agonism, Toll-like receptor ligands, and homeostatic cytokines such as IL-4 or IL-21 (138). Some studies have used CD27^+^ memory B cells (139). However, sorting Ag-experienced B cells (via 4-1BBL), and endowing them with potent APC function, can serve as a unique tool in B cell–based therapies.

In the preclinical glioma model CT2-A, repeated administration of B_Vax_ and anti–PD-L1 allowed adoptively transferred CD8^+^ T cells to eradicate the tumor and prevent its regrowth upon reinjection in the opposite hemisphere in 50% of the treated mice after brain radiation and temozolomide treatment (GBM patient standard of care). Tumor eradication correlated with prominent infiltration of CD8^+^ T cells in the tumor cell injection sites. CD8^+^ T cells were also found in the choroid plexus, a structure that plays a fundamental role in CNS immunosurveillance via the cerebrospinal fluid–brain barrier (140). However, CD8^+^ T cells were also present in more distant sites like the cerebellum and pons, suggesting organ-wide surveillance to protect the CNS. Accordingly, CNS-infiltrating CD8^+^ T cells show an activated phenotype (characterized by the expression of IFN-γ and CD44) and the absence of inhibitory molecules such as PD-1 or TIGIT. These findings suggest that fully functional memory-like CD8^+^ T cells persist in the target organ. GBM patient–derived B_Vax_ is generated from the patient’s peripheral blood. This study used freshly resected patient tumors as a protein homogenate (tumor lysate) source. B_Vax_ were incubated with tumor lysate and tested for the ability to activate autologous CD8^+^ T cells in the absence of exogenous TCR stimulation. CD8^+^ T cells cultured with B_Vax_ pulsed with tumor lysates obtained from the same patient significantly expanded granzyme B–expressing CD8^+^ T cell numbers. This observation was almost exclusive to B_Vax_ pulsed with autologous tumor. We tested activated and expanded CD8^+^ T cells’ ability to kill autologous tumor cells via in vitro cytotoxicity assay. The results showed that CD8^+^ T cells activated via B_Vax_ potently kill glioma cells while sparing nontumor cells, in both newly diagnosed GBM and recurrent GBM biospecimens. These results support human B_Vax_ as promoting anti-GBM autologous CD8^+^ T cell activity.

**Figure 3:**
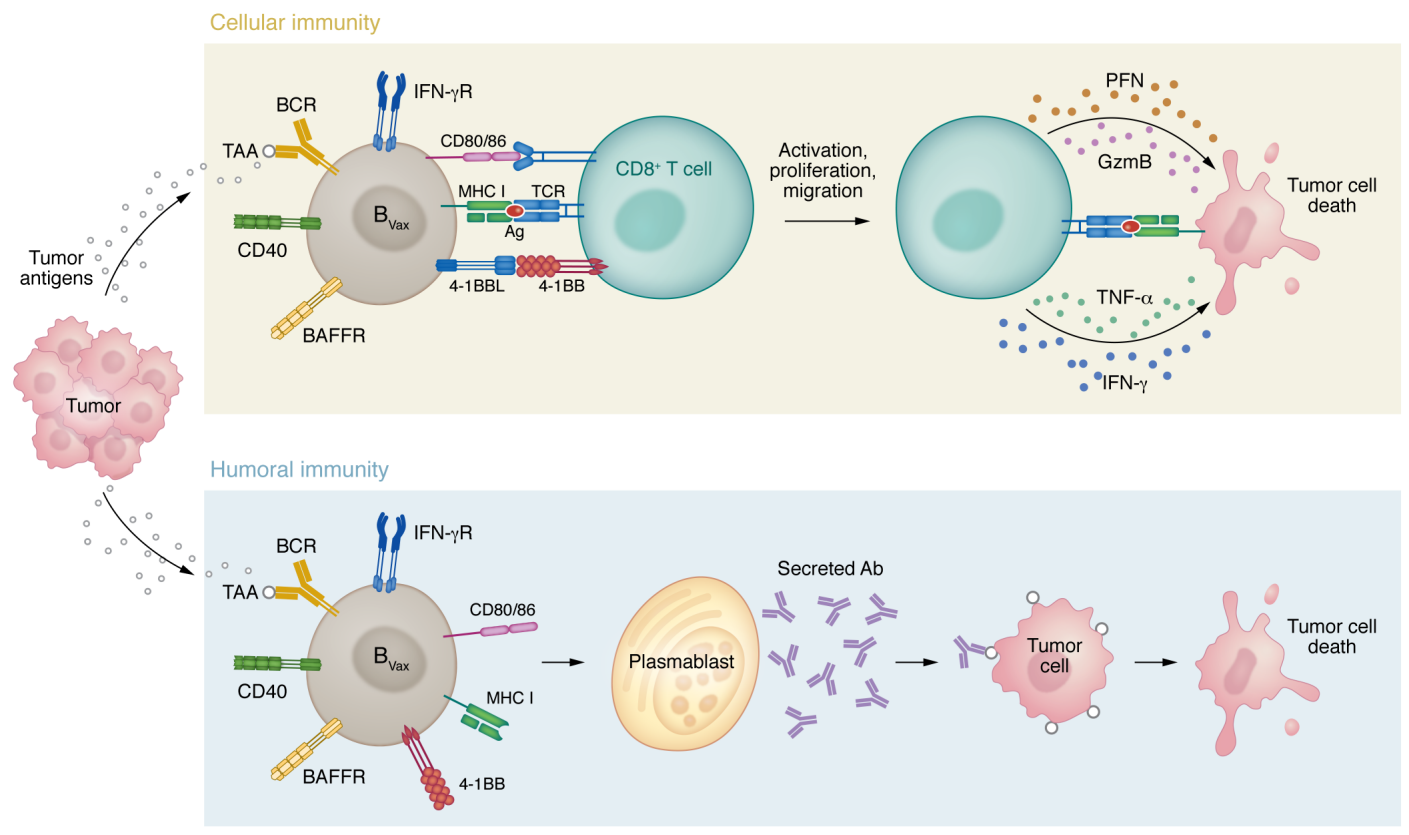
B cell vaccine immune effector functions. B_Vax_ can exert an antitumor immune response via cellular immunity (activation of CD8^+^ T cells) and humoral immunity (production of tumor-reactive antibodies). PFN, perforin.

In addition to the APC function, B_Vax_ differentiates into plasmablasts and produces tumor-reactive antibodies with therapeutic potential. While further studies are needed to elucidate the exact reactivity of B_Vax_-derived IgG and its effector immune functions, it is undeniable that B_Vax_ represents a unique immunotherapy platform that merges both cellular (CD8^+^ T cell activation) and humoral (Ab production) function. Thus, the effectiveness of our approach relies on both cellular (CD8^+^ T cell activation) and humoral (Ab production) antitumor immune processes. These effector functions are unexplored in the brain tumor field and underexplored in cancer research in total. Figure 3 summarizes therapeutic B_Vax_ effector immune functions.

## Conclusions

APC therapy in GBM was initiated more than two decades ago with the development of DC vaccines. Even though the therapeutic effect of this approach is inconclusive, a wealth of promising preclinical results urges us to continue developing APC therapies to boost the antitumor immune response, including novel cellular choices, such as B cells. The choice of the source and nature of the antigen, paired with tools to fight the glioma’s immunosuppressive microenvironment, is a key factor to be considered for future approaches. Treating preoperatively metastatic breast cancer and non–small cell lung cancer with ICI has proved that antitumoral immune responses can be generated if immunotherapy is administered while the tumor (and possibly draining lymph nodes) is present. Immunological response correlated with high rates of pathological response and improved long-term survival. Therefore, one might consider that the potential immunological impact of maximum versus partial resection, or even neoadjuvant immunotherapy treatment, in newly diagnosed GBM could improve immune therapies’ clinical outcomes.

## Figures and Tables

**Figure 1:**
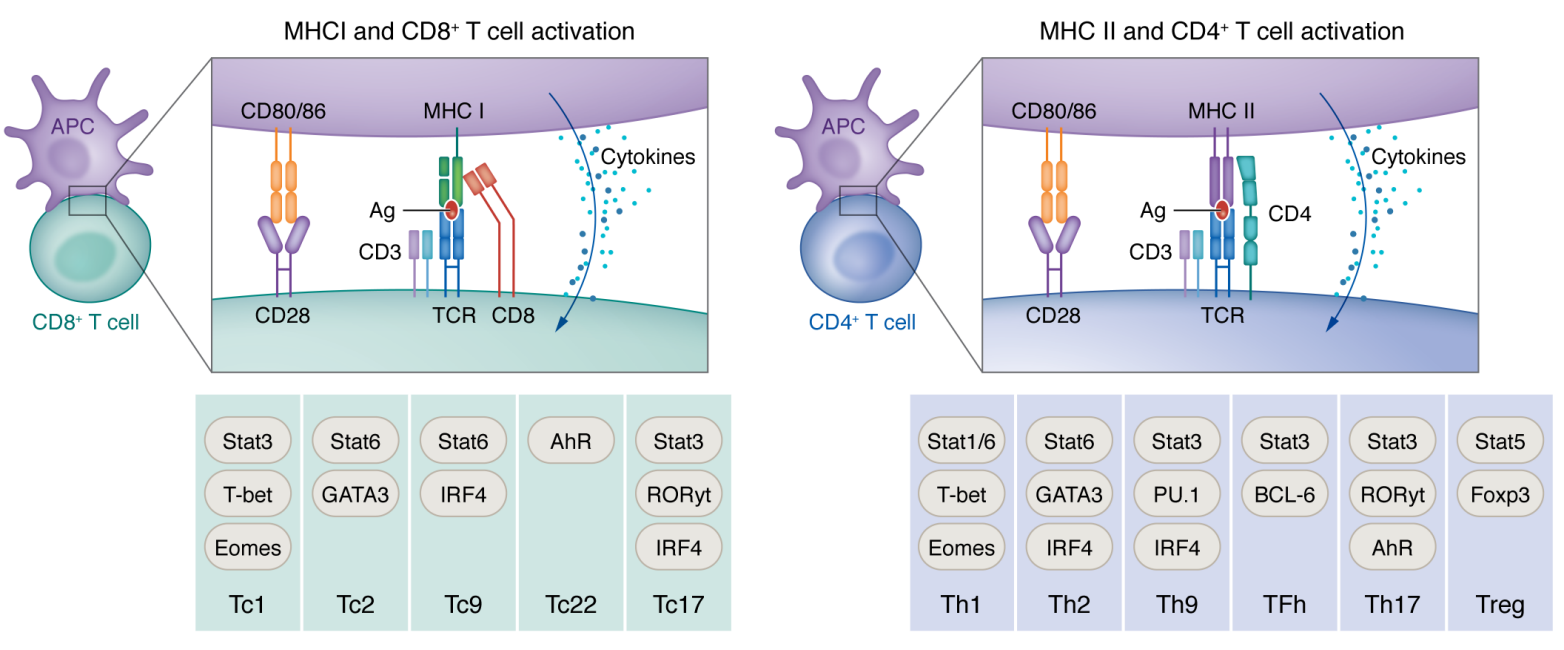
MHC-dependent antigen presentation. CD8^+^ (left) and CD4^+^ (right) T cell receptors are activated via antigens presented by MHC I and MHC II, respectively (first signal). Together with the costimulatory signal through CD28 engagement (second signal) and cytokines (third signal), this machinery can activate different T cells into different functional subsets (bottom rows of figure). For more details on naive CD4^+^ and CD8^+^ T cell differentiation and effector functions, see reviews (6, 141). AhR, aryl hydrocarbon receptor; BCL-6, B cell lymphoma 6; Eomes, eomesodermin; Foxp3, forkhead box P3; GATA3, GATA-binding protein 3; IRF4, interferon-regulatory factor 4; ROR, retinoic acid receptor–related orphan receptor; Tc, cytotoxic T cells; TCR, T cell receptor; Tfh, T follicular helper.

**Table 2:**
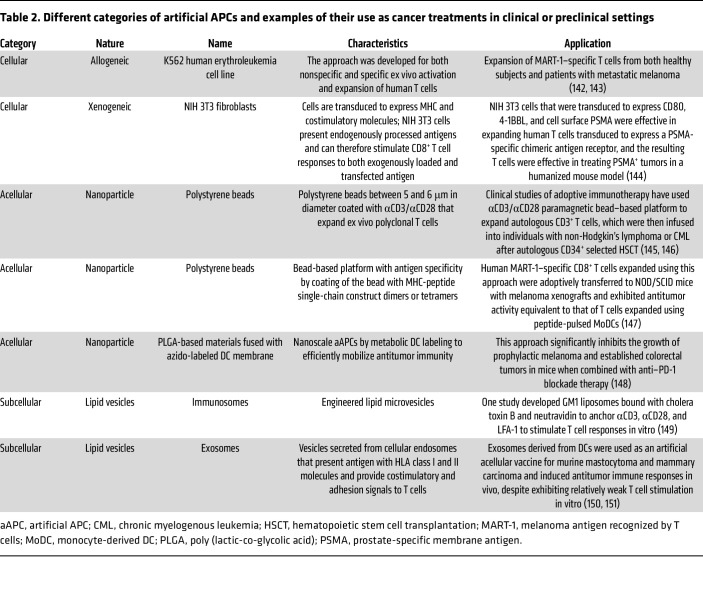
Different categories of artificial APCs and examples of their use as cancer treatments in clinical or preclinical settings

**Table 1:**
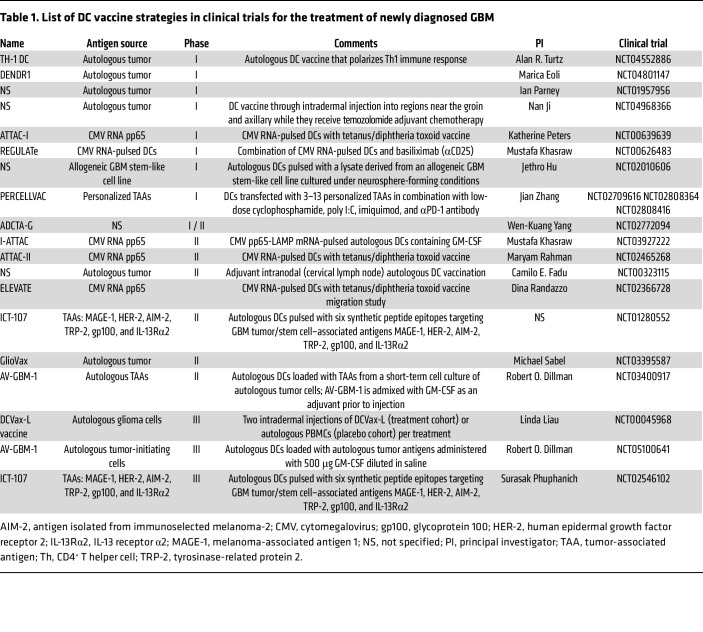
List of DC vaccine strategies in clinical trials for the treatment of newly diagnosed GBM

## Version 1 02/01/2023

Electronic publication
